# Atomistic Structure of Mineral Nano-aggregates from Simulated Compaction and Dewatering

**DOI:** 10.1038/s41598-017-15639-4

**Published:** 2017-11-10

**Authors:** Tuan Anh Ho, Jeffery A. Greathouse, Yifeng Wang, Louise J. Criscenti

**Affiliations:** 1 Geochemistry Department, Sandia National Laboratories, Albuquerque, New Mexico, 87185 USA; 2 Nuclear Waste Disposal Research and Analysis Department, Sandia National Laboratories, Albuquerque, New Mexico, 87185 USA

## Abstract

The porosity of clay aggregates is an important property governing chemical reactions and fluid flow in low-permeability geologic formations and clay-based engineered barrier systems. Pore spaces in clays include interlayer and interparticle pores. Under compaction and dewatering, the size and geometry of such pore spaces may vary significantly (sub-nanometer to microns) depending on ambient physical and chemical conditions. Here we report a molecular dynamics simulation method to construct a complex and realistic clay-like nanoparticle aggregate with interparticle pores and grain boundaries. The model structure is then used to investigate the effect of dewatering and water content on micro-porosity of the aggregates. The results suggest that slow dewatering would create more compact aggregates compared to fast dewatering. Furthermore, the amount of water present in the aggregates strongly affects the particle-particle interactions and hence the aggregate structure. Detailed analyses of particle-particle and water-particle interactions provide a molecular-scale view of porosity and texture development of the aggregates. The simulation method developed here may also aid in modeling the synthesis of nanostructured materials through self-assembly of nanoparticles.

## Introduction

The structure of clay aggregates (i.e., porosity, pore geometry, and pore size distribution) under subsurface conditions is one of the important factors controlling gas/oil in place, ion exchange capacity, hydrologic conductivity, and mechanical strength of low-permeability geologic formations^1^. Clay minerals are composed of sheets of metal ions either tetrahedrally (T) or octahedrally (O) coordinated by framework oxygen ions or hydroxyl groups. Pore spaces in clay-bearing rocks include interlayer [i.e., space internal to the particle between clay layers (i.e., TOT layers)] and interparticle (i.e., space between clay particles or platelets) pores^2,3^. Interparticle porosity decreases under low and moderate compaction, while interlayer porosity only decreases under large compression and depends on the amount of water present in the interlayer pores (i.e., 1 or 2 water layers)^4^. The exchange of chemical species including water, gases and ions between interlayer and interparticle pores is vital to understanding clay swelling^5,6^, the migration of radionuclides away from a nuclear waste disposal site^7^, and the potential release of CO_2_ through shale caprocks in a subsurface carbon storage facility^8,9^. The migration of fluids in interparticle pores particularly affects oil and gas extraction. For example, in shale plays, gas flows from the shale matrix dominated by micropores to an interconnected fracture system^10^.

The variation of water content (i.e., dry, partially saturated, and saturated) in clay pores determines in part the partitioning of ions between the bulk aqueous solution in larger pores and the adsorbed species on clay basal and edge surfaces^11,12^. The amount of water in clay aggregate also influences the adsorption of industrially important gases such as CH_4_ and CO_2_^13^. Dewatering of clays during sedimentation also affects mudstone porosity: the more rapidly deposited the clay platelets, the less time the interparticle pores have to dewater, and therefore the water content remains high^14^. While the effects of water on interlayer properties and clay swelling are well studied^15^, the effects of dewatering on clay aggregate structure at the nanoscale under sedimentation are largely unexplored^16^. Here we investigate the change in micro-porosity of clay-like nanoparticle aggregates under relevant sedimentation/diagenesis conditions using molecular modeling techniques.

From a classical molecular dynamics (MD) simulation perspective, designing clay aggregates for modeling is challenging. The first challenge is the system size, particularly how to use nanoparticles to provide a reasonable representation of actual clay particles found in nature, with particle sizes (e.g., microns) beyond the existing computational capability. The second challenge for modeling clay particles is that the structure of edge surfaces of clay particles under varying pH conditions is not well understood^17^. Numerous quantum mechanical simulations tried to address this issue by studying the stability^17,18^ and acid-base properties of clay edge surfaces^19–22^. At the molecular level, one of the most widely used force fields to describe water-clay mineral systems (i.e., ClayFF^23^) was initially developed to simulate the interaction of water with unreactive basal surfaces and interlayers of layered minerals, but the applicability of ClayFF to edge surfaces has only recently been investigated^24–26^. Consequently, the majority of MD simulation studies of clay-water systems have been carried out using a simple slit-shape pore without considering edge effects^13,15,17,27–32^. Lammers *et al*.^29^ attempted to simulate clay particles and observed the detachment of OH groups from particle edges. Two studies have simulated ion and water transport between interparticle and interlayer regions of smectite particles including the edge surfaces, but only orthogonal-shaped pores were included along with fully rigid clay layers^2,33^. Although interesting results were obtained from these studies, a complex and realistic clay aggregate model with interlayer and interparticle pores, and interparticle boundaries at the atomistic scale is required to fully investigate the exchange of water and ions between interlayer and interparticle pores^34^. Simulations of increasingly complex pore sizes and geometries can be used to study, for example, the relative significance of ion adsorption onto the basal versus edge surfaces of clay minerals, the effect of nanoscale fluid flow on the metamorphism of the continental crust^35^, and properties of nanoporous organic matter in shale^36^.

In this paper, we develop a methodology to create nanoscale models of multi-particle, dual porosity clay aggregates based on the endmember layered mineral gibbsite, Al(OH)_3_, in order to simulate more “rock-like” systems and to be able to characterize the coupling of transport and chemistry in these systems. This work represents a breakthrough in the preparation of simulation models of nanoporous materials in which the pore structure depends on the compaction and dewatering processes. Such models can then be used to simulate nanopore properties in both natural and engineered materials. Our example is based on nanoparticles comprised of a layered mineral, but the methodology could be applied to virtually any type of nanoparticle composition or geometry.

## Results

### Impact of dewatering rate on the stacking of clay-like nanoparticles

**Figure 1:**
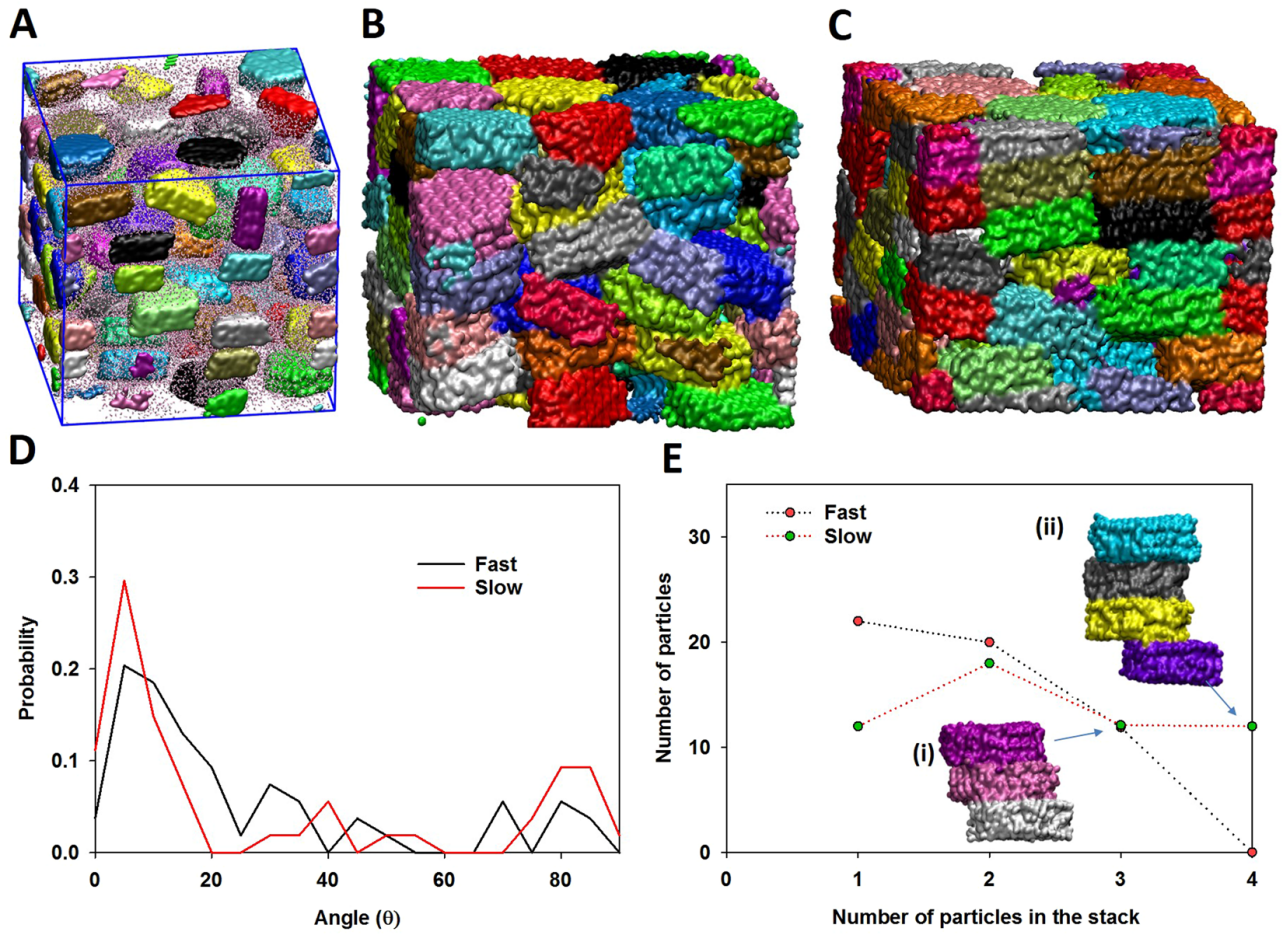
Simulation snapshot representing the hydrated clay-like nanoparticle aggregates that we used as a starting system to study the effects of dewatering and water content on aggregate structure **(A)**. Simulation snapshots showing the effect of *‘fast’* **(B)** and *‘slow’* **(C)** dewatering rate on dry aggregate structures. Comparison of particle orientations **(D)** and the stacking of particles **(E)** under different dewatering rate. Insets (i) and (ii) illustrate stacks of 3 and 4 nanoparticles, respectively.

In Fig. 1 we present the results to demonstrate the effects of dewatering on the particle-particle interaction and particle alignment. The micro-porosity and texture of nanoparticle aggregates largely depend on the manner that clay-like particle organized. Starting with the hydrated gibbsite aggregate shown in Fig. 1A (see Method for details generating this system), water molecules were randomly removed from the simulation cell while the system was compressed (P = 100 MPa, T = 300 K) until the system reached an equilibrium state (i.e., constant volume). For *‘fast’* dewatering, all water molecules were completely removed at the beginning of the simulation. For *‘intermediate’* dewatering rate (results not shown in Fig. 1), 100 water molecules were withdrawn from the cell every 100 time steps (no waters remained in the simulation box after 55 ps). For *‘slow’* dewatering, 10 water molecules were randomly deleted every 100 steps (no waters remained in the simulation box after 550 ps). Visual inspection of the simulation snapshots indicates that particles formed during ‘*slow*’ dewatering processes (Fig. 1C) form large stacks with close contacts between basal surfaces (i.e., bulk-like) to form a more compact system compared to ‘*fast*’ dewatering (Fig. 1B). During ‘*fast’* dewatering (Fig. 1B), less stacking of nanoparticles results in larger pores. To quantify this observation we calculated the orientation of particles (Fig. 1D) and the number of particles that form stacks of 2, 3, or 4 particles for fast and slow dewatering (Fig. 1E). In Fig. 1D, θ is the angle between the vector normal to the basal surface of each particle and the z direction of the simulation box. In general, the results in Fig. 1D indicate the preferred orientation of nanoparticles (i.e., parallel to each other), which agrees with experimental data^16,37^. For *‘slow’* dewatering, there is higher possibility that the particles are aligned parallel to the *xy*-plane compared to *‘fast’* dewatering. As more particles are oriented nonparallel to the *xy*-plane, the porosity increases. As shown in Fig. 1E, for *‘fast’* dewatering approximately 22 out of 54 nanoparticles do not stack on top of another particle. These delaminated particles are responsible for the high porosity in the aggregates. During *‘slow’* dewatering, aggregates of 3 or 4 nanoparticles form (see inset ii) but during *‘fast’* dewatering the largest aggregates contain only 3 nanoparticles.

### Impacts of dewatering rate and water content on nano-structural properties of aggregates

**Figure 2:**
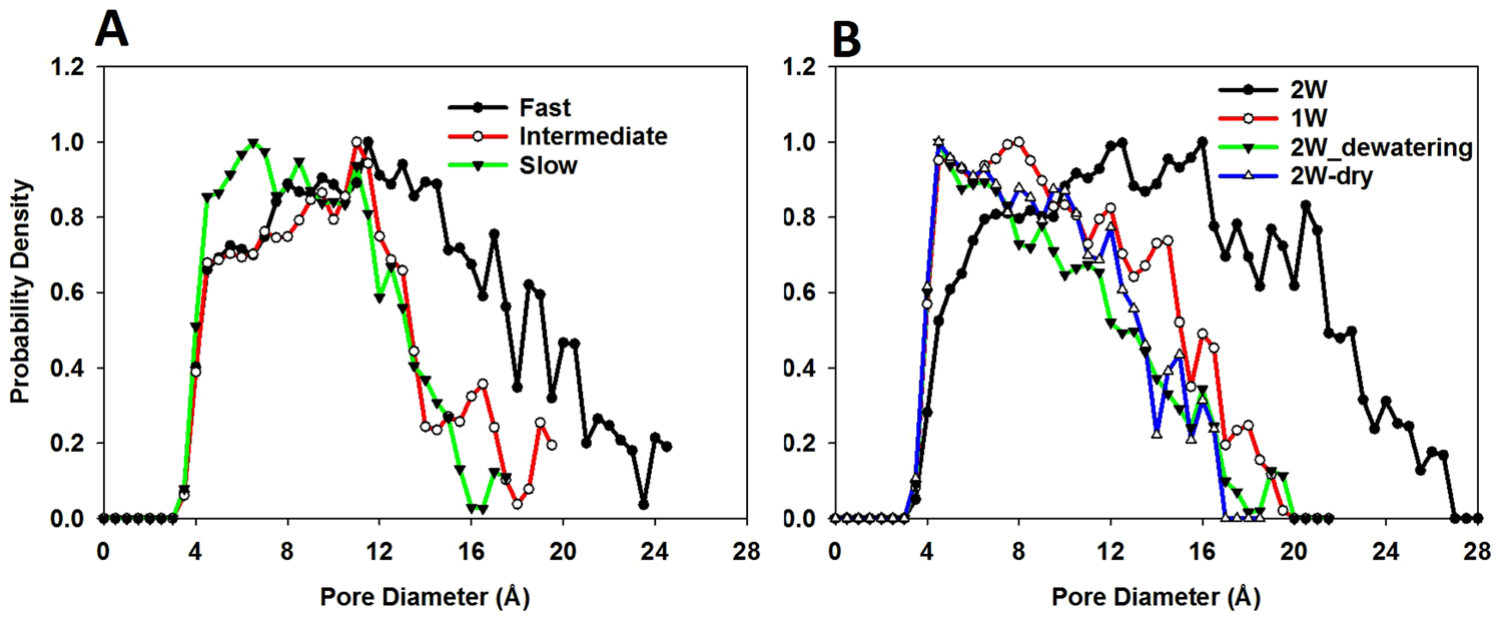
Effect of dewatering rate (**A**) and water content (**B**) on the pore size distribution of nanoparticle aggregates.

In Fig. 2A we summarize the effect of dewatering rate on the pore size distribution (PSD) calculated for clay-like nanoparticle aggregates. The PSD was calculated by using the method proposed by Gubbins *et al*.^38^. The results indicate that the PSD varies significantly depending on the dewatering process. *‘Fast’* dewatering (black line-Fig. 2A) yields a wider PSD with larger pores developed, compared to slower dewatering. For *‘fast’* dewatering, the pores in the aggregate include micropores (<2 nm^39^) and mesopores (2–50 nm^39^); for the *‘intermediate’* and *‘slow’* dewatering processes, only micropores are formed. One possible explanation for this difference is that under fast dewatering and compaction, nanoparticles do not have enough time to rearrange themselves into a large stack, which would result in larger pores in the aggregate structure.

To study the effect of water content on the clay-like nano-aggregate, from the configuration of Fig. 1A different nanoparticle-water systems with various amounts of water were created. In the first system, all water molecules farther than 3 Å away from any nanoparticle were removed at the beginning of the simulation. This method created a nanoparticle-water system in which each particle is surrounded by a 3 Å water film (‘*1 W*’), and the total water content is 22.5 wt%. In the second system, each particle is surrounded by a 6 Å water film *(‘2 W’*), and the water content is 37.2 wt%. These systems were then compressed at P = 100 MPa, T = 300 K until an equilibrium state (i.e., constant volume) was achieved. The *‘1 W’* and *‘2 W’* cases represent the closed systems where water exchange with the surrounding environment does not occur. To explore the possibility that water can be expelled from the system during compression^37^, in a third system all water molecules that moved into a predefined region during the simulation of the *‘2 W’* system were extracted from the system (Method). Note that water molecules can only move into the predefined region if they can desorb from the nanoparticle surfaces and if there is pore connectivity. This extraction method was described in more detail in our previous study of methane release in kerogen nanopores^40^. This simulation was conducted for 89 ns, during which the water content was reduced from 37.2 wt% (i.e., that of *‘2 W’* system) to 6 wt%. The results obtained for this system are labeled as *‘2W_dewatering’*. Note that after 89 ns, water molecules were still able to move to the predefined region but at a very slow rate (i.e., for the last 1 ns, only 13 water molecules were deleted). Assuming that all water molecules would eventually be expelled from the system, we deleted the remaining water molecules and continued compressing the simulation box. The results reported for this system are labeled as *‘2 W_dry’*.

As shown in Fig. 2B, water content in the aggregates also affects the PSD. For example, in the *‘2W’* system, pore diameters can be as large as 26 Å. Lower water content leads to smaller pore sizes in the aggregates. In the*‘1 W’* system, only pores smaller than 20 Å are observed. In addition, the maximum in the pore size distribution profile is found from 12 to 16 Å for the*‘2 W’* system while for other systems (i.e., ‘*1 W’*, ‘*2W_dewatering’*, ‘*2W_dry’*) the maximum is observed from 4 to 8 Å. This indicates that the pore structure and pore size in the clay aggregates vary depending on the abundance of water. Pores in the hydrated nanoparticle aggregates are larger than those in the dry aggregates (‘*2W_dewatering’*, ‘*2W_dry*’). Note that pore spaces in the*‘1 W’* and *‘2 W’* systems are completely filled by water.

Our results also suggest that early dewatering and compression might play a dominant role in the pore size distribution of the gibbsite aggregates. A similar pore size distribution was found in the ‘*slow*’ system in Fig. 2A and the ‘*2W-dry*’ system in Fig. 2B (both systems are dry and obtained at different dewatering rates, it takes 0.55 ns for the ‘*slow’* system, and more than 89 ns for the *‘2W-dry’* system to be dried). This indicates that late dewatering and compression (e.g., after 0.55 ns in our simulations) does not significantly affect the pore structure of nanoparticle aggregates. Both early compression and dewatering (less than 0.55 ns) significantly affect pore structures.

**Figure 3:**
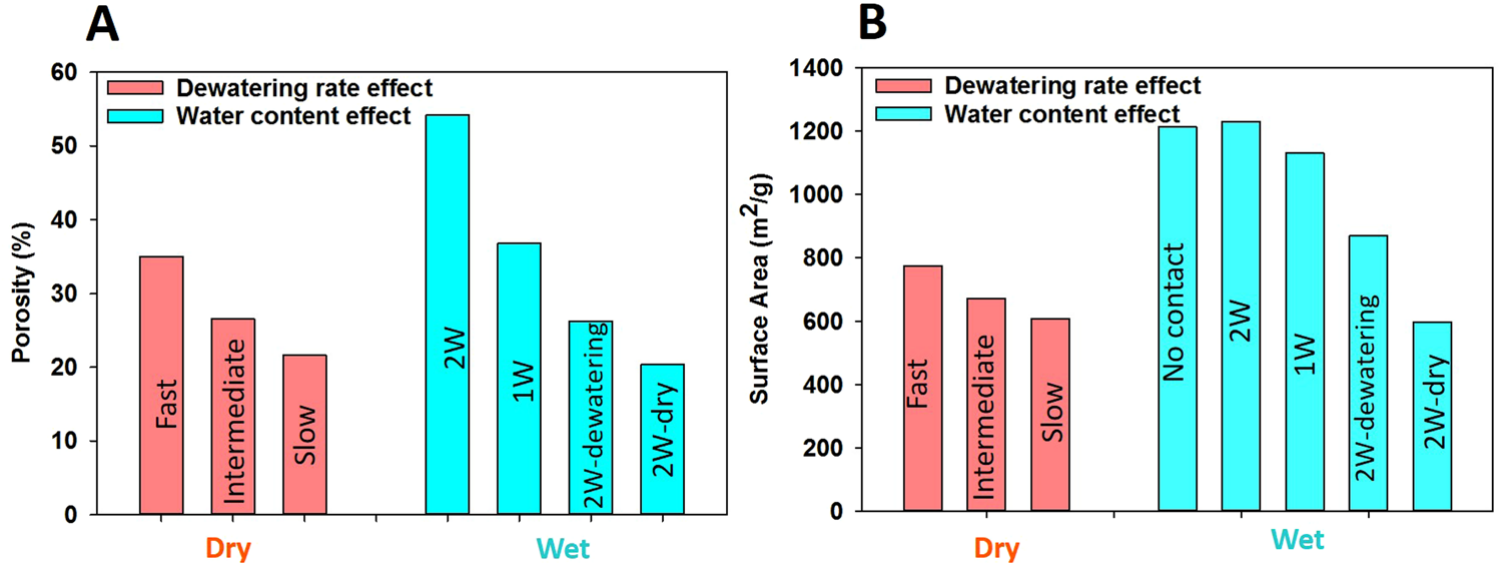
Effect of dewatering rate and water content on porosity (**A**) and surface area **(B)** of nanoparticle aggregates.

In Fig. 3 we illustrate the effects of dewatering rate and water content on the total porosity and pore surface area (SA) of the nanoparticle aggregates. The porosity and surface area were calculated using Materials Studio software^41,42^. Note that the SA reported in Fig. 3B is larger than that observed for gibbsite (i.e., 418 m^2^/g)^43^ and typical clays (i.e., ~30 m^2^/g)^10^ because of the small particle size in our simulations. The results suggest that decreasing the dewatering rate and water content reduces the porosity of nanoparticle aggregates. The reason for this observation can be explained via the SA data presented in Fig. 3B. When the accessible SA decreases, the number of particle-particle contacts increases, and therefore the porosity decreases. The large accessible SA obtained for *‘fast’* dewatering indicates that particle-particle contact is not preferred, compared to slower dewatering systems. This is because particles do not have enough time to arrange and organize to maximize their contact area. When particles are surrounded by thin water films (i.e., ‘*2 W’* and *‘1 W’* systems) there is almost no direct particle-particle contact (i.e., surface area for *‘2 W’* and *‘1 W’* is comparable with the surface area when there is no contact between particles). When the amount of water is reduced, the number of particle-particle contacts increases, leading to a decrease in porosity.

**Figure 4:**
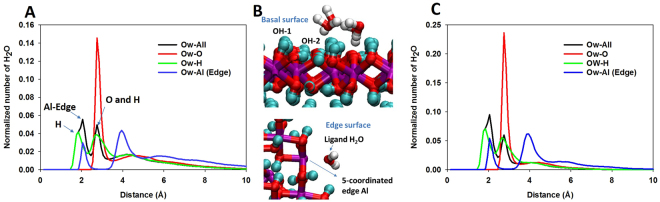
Relative position of water O atom (Ow) with respect to gibbsite nanoparticle atoms for 2 W **(A)** and 1 W **(C)** systems. Simulation snapshots demonstrate the interaction of water with different sites on gibbsite basal and edge surfaces **(B)**. Red, pink, and cyan spheres represent for O, Al, and H atoms of gibbsite, respectively. Red and white spheres represent water O and H atoms, respectively. In (**A**) and (**C**), red, green, and blue lines represent the closest distance of Ow atoms to O, H, and edge-Al atoms of the nanoparticles in the aggregate, respectively, while the black line represents the closest distance of Ow atoms to particle atoms.

Water structure at the nanoparticle surfaces is strongly dependent on water content. Fig. 4A and C illustrate the density profiles of water oxygen atoms near nanoparticle surface atoms for the *‘1 W’* and *‘2 W’* systems. For the *‘2 W’* system, the density profile (black line) indicates that water oxygen atoms (O_w_) form a first peak around 2 Å with a shoulder at approximately 1.75 Å from the particle atoms. A second peak is found at 2.75 Å. Detailed analysis indicates that the shoulder peak at 1.75 Å is a result of the interaction between O_w_ atoms and H atoms of the gibbsite particles (i.e., the shoulder peak for O_w_-all aligns with the first peak in the O_w_-H density profile). Simulation snapshots in Fig. 4B show that these hydrogen atoms are mainly the hydrogen atoms of OH groups that point away from the surface (denoted OH-1 on Fig. 4B) and possibly the OH groups at the edge (not shown). The peak at 2.0 Å is associated with the accumulation of coordinating water molecules (Fig. 4B) near (5-coordinated) Al atoms at the edge (i.e., this peak is aligned with the first peak on the density profile of Ow-Al(Edge)). The adsorption of a water molecule to form a complete octahedral (6-coordinated) shell about each edge Al atom is expected^24^. The second peak at 2.75 Å is associated with the interaction of O_w_ with the surface hydroxyl O and H atoms denoted OH-2 in Fig. 4B (note that the OH-2 groups are almost parallel to the gibbsite surface). Similar peak positions were observed at lower water content (Fig. 4 C). However, because of the change in the porosity and pore size distribution when water content was reduced from *‘2 W’* to ‘*1 W*’, the majority of water in the ‘*1 W’* system is interfacial water (i.e., the peak intensities for the ‘1 W’ system are higher than for the ‘2 W’ system). In addition, a significant fraction of water molecules in the ‘*2 W’* system can be considered bulk-like since they are located between 8 to 10 Å from the nanoparticle surface (see Fig. 4A).

## Discussion

In this work, we have built a model gibbsite nanoparticle and have used MD simulations to study the structure and texture of aggregates formed by the nanoparticles. We found that dewatering and water content affect the porosity and structure of aggregates formed during compaction. Fast dewatering yields larger pores and a wider pore size distribution, compared to slower dewatering. When water is abundant in the system, gibbsite nanoparticles were surrounded by water that prevented direct particle-particle contact, resulting in a higher porosity. The particle-particle and water-particle interactions are responsible for the origin of aggregate porosity during compression. If particles orient and arrange to maximize the interactions between gibbsite basal surfaces, the aggregate will have lower porosity. Porosity from our simulations agree with available experimental data despite the fact that our model nanoparticle is much smaller than those formed in nature or by synthesis.

Our work provides insight into aggregate micro-porosity based on layered Al(OH)_3_ (gibbsite) nanoparticles, including the effects of compaction and dewatering processes. Subsequent MD simulations will probe the structural and dynamic properties of hydrated clay mineral aggregates under much more diverse pore environments than is usually reported. Additionally, we establish a methodology for creating more realistic and complex nanoparticle systems of varying porosity, which can be extended to other types of nanoparticles created from bulk structures. For example, the simulation method developed here may also help model the synthesis of nanostructured materials through self-assembly of nanoparticles^44^. Finally, the aggregate in this work is created with individual particles of the same size and shape. Future work will need to investigate the effects of particle polydispersity and size/shape distribution on the final pore size distribution and compaction of clay aggregates^45,46^.

## Method

We first describe our method for constructing a gibbsite nanoparticle with an explicit representation of basal and edge surfaces. Then we describe our approach to build nanoparticle aggregates.

### Single nanoparticle

We selected gibbsite [Al(OH)_3_] as a good starting point for developing multi-particle simulation models because its structure consists of stacked sheets of linked octahedra of aluminum hydroxide. These neutral aluminum hydroxide sheets are similar to the octahedral sheets bonded to silicate sheets in clay minerals such as kaolinite, illite and smectite. Our choice of gibbsite permits us to design particle aggregates similar in shape to clay particles and with interparticle porosity only; the complexity associated with interlayer porosity in clay platelet aggregates will be addressed in future work.

**Figure 5:**
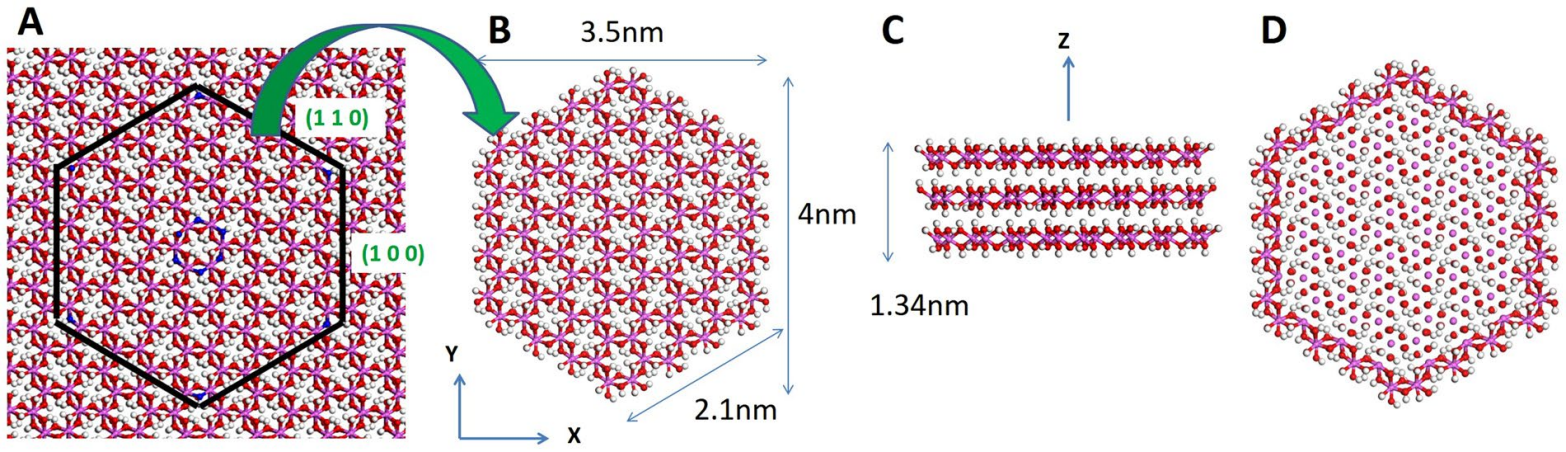
Gibbsite nanoparticle construction. Red, white, and pink spheres represent oxygen, hydrogen, and aluminum atoms, respectively. (**A**) Basal gibbsite surface with pre-defined cutting directions (black). Atoms colored blue at the center and corners of the hexagon guide the cleavage. (**B**) Hexagonal gibbsite nanoparticle and its dimensions. (**C**) Three layers of gibbsite nanoparticle. (**D**) All Al-O-Al angles at the particle perimeter were defined in our simulations in addition to the ClayFF force field.

Each gibbsite layer consists of a sheet of octahedral Al atoms coordinated by 3 hydroxyl O atoms each above and below the Al sheet. Gibbsite grows well along the lateral (‘*a*’ and ‘*b*’) directions that include the octahedral sheets, resulting in thin hexagonal nanoparticles^43,47–49^. In our approach, a hexagonal gibbsite particle was cleaved from a slab of 3 layers of gibbsite along the (1 0 0) and (1 1 0) directions as shown in Fig. 5A. The (1 0 0) and (1 1 0) faces were selected since they are the most common for particles with a pseudo-hexagonal shape^50^, and these faces are also observed experimentally for gibbsite nanoparticles^48^. Additional OH groups were added to coordinate edge Al atoms, resulting in five-fold coordination of edge Al atoms. On the basal (0 0 1) surface there are only bridging OH groups (i.e., OH group bonded to 2 Al atoms), but on the edge surfaces there are both singly-coordinated (i.e., OH group bonded to 1 Al atom) and bridging OH groups. The structure of the basal and edge surfaces in our model is consistent with experimental data^48^.

Each hexagonal nanoparticle has 2016 atoms including 288 Al, 864 O, and 864 H atoms, 2.1 nm edges (Fig. 5B), and a thickness of 1.34 nm (3 gibbsite layers, Fig. 5C). This particle is much smaller than those synthesized in the laboratory or found in the nature^43,47–49^. However, this work focuses on the micro-porosity of nanoparticle aggregates, and in consideration of computational resources and simulation time, our particle size selection is a reasonable choice. Coarse grained techniques^51^ can be used to simulate larger particles and system sizes, but the interfacial chemistry is not well described at this level of theory.

The total potential energy of the gibbsite nanoparticle is described using ClayFF, in which interatomic interaction energies are described by nonbonded Lennard Jones and electrostatic energy terms^23^. Partial charges of +1.575 e, −0.95 e, and +0.425 e are assigned to the Al, O, and H atoms, respectively, which results in a charge-neutral particle. The only bonded energy term used in ClayFF for the mineral phase is the OH bond stretch. ClayFF was first developed to simulate clay mineral basal surfaces with periodic boundary condition (*i*.*e*., no edges). Recent efforts further developed ClayFF by defining harmonic Mg-O-H and Al-O-H angle bending terms to simulate specific edges of brucite and gibbsite, respectively^24,25^. Although the (1 0 0) edge can be successfully simulated using the new Al-O-H angle bending term, a hexagonal gibbsite particle with (1 0 0), (1 1 0), and (0 0 1) surfaces was not numerically stable. To overcome this issue, an Al-O-Al angle bending term was explicitly defined using a harmonic potential for the Al-O-Al angles at the edge surfaces (Fig. 5D). In our simulations, equilibrium values for Al-O-H and Al-O-Al angles are 110° and 100°, respectively. Because particle-particle interactions and collisions in our multi-particle simulations discussed below are quite strong, we used *k* = 800 kcal.mol^−1^.rad^−2^ to maintain the nanoparticle edge structure. Note that the use of a large *k* value will affect the calculated vibrational properties, but it is unlikely to affect the structural properties of nanoparticle aggregates reported in this work.

### Nanoparticle aggregates

**Figure 6:**
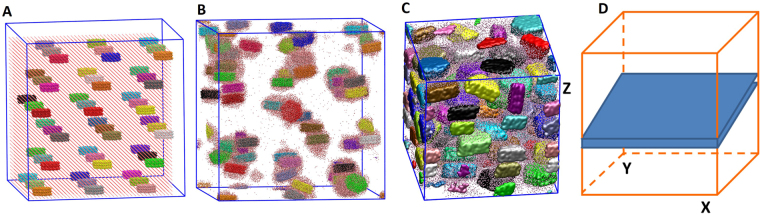
Snapshots represent configurations at different stages of simulations as described in the text: initial configuration of nanoparticles and water **(A)**, after initial NVT simulation **(B)**, and after initial NPT simulation **(C)**. Water molecules are presented by red-white wireframes. Nanoparticles are illustrated by different isosurfaces representing the van der Waals radii of perimeter atoms. A schematic shows a slab region (blue) of 10 Å thickness in the z direction that we used to study the water expulsion from the simulated system during compression (**D**).

To construct nanoparticle aggregates, 54 gibbsite nanoparticles were initially placed in a simulation box of 30 × 30 × 30 nm^3^ (Fig. 6A) followed by the addition of 55,008 water molecules. Initial water-water and water-nanoparticle distances were 7 Å and 8 Å, respectively (Fig. 6A). This system was equilibrated for 0.3 ns in the NVT ensemble (constant number of particles, volume, and temperature T = 300 K) using LAMMPS^52^. During this simulation, long-range electrostatic interactions were calculated using PPPM (particle-particle-particles-mesh) solver^53^ with a time step of 1 fs. The simulation temperature was kept constant by applying a Nosé-Hoover thermostat^54^. The rigid SPC^55^ model was used for water, along with the SHAKE algorithm^56^. The use of rigid SPC^55^ or flexible SPC^57^ water model will not affect the structural properties presented herein. However, when studying spectroscopic and other dynamic properties that vary with changes in molecular geometry, the flexible SPC water model should be implemented^58^. Interactions among unlike atom types were calculated using an arithmetic mixing rule. After initial equilibration as described above, the system (Fig. 6B) was compressed using an NPT ensemble (constant number of particles, pressure 100 MPa, and temperature 300 K) simulation until the simulation cell volume reached a constant value (Fig. 6C). During this initial NPT simulation, long-range electrostatic interactions were turned off. A cut-off distance of 10 Å was used for all the non-bonded interactions. The final configuration illustrated in Fig. 6C is the system that we used to study the effects of dewatering and water content on the structure and porosity of nanoparticle aggregates. For all subsequent dewatering simulations, long-range electrostatic interactions were once again calculated with PPPM.

#### Effect of dewatering rate on aggregate volume and texture

Starting with the hydrated gibbsite aggregate shown in Fig. 6C, three new simulations were performed to investigate the impact of evaporation or dewatering rate on the aggregate structure and porosity. In each simulation, water molecules were randomly removed from the simulation cell while the system was compressed under NPT conditions (P = 100 MPa, T = 300 K) until the system reached an equilibrium state (i.e., constant volume). In the first system, all water molecules were completely deleted at the beginning of the simulation. In the second system, 100 water molecules were withdrawn from the cell every 100 time steps (no waters remained in the simulation box after 55 ps). In the third system, 10 water molecules were deleted every 100 steps (no waters remained in the simulation box after 550 ps). The results reported for the first, second, and third systems are labeled as ‘*fast’*, ‘*intermediate’*, and ‘*slow’*, respectively. The range of dewatering rates in this work is arbitrarily selected because of the unknown dewatering rate in the process of sedimentation. However, the method could be applied for a much slower rate using enhanced simulation techniques, but our initial results presented herein already demonstrate the effect on aggregate structure with only minor changes to the dewatering rate.

#### Effect of water content on aggregate volume and texture

From the configuration of Fig. 6C different nanoparticle-water systems with various amounts of water were created. In the first system, all water molecules farther than 3 Å away from any nanoparticle were removed at the beginning of the simulation. This method created a nanoparticle-water system in which each particle is surrounded by a 3 Å water film (‘*1 W*’), and the total water content is 22.5 wt%. In the second system, each particle is surrounded by a 6 Å water film *(‘2 W’*), and the water content is 37.2 wt%. These systems were then compressed under NPT conditions (P = 100 MPa, T = 300 K) until an equilibrium state (i.e., constant volume) was achieved.

In the *‘1 W’* and *‘2 W’* systems, the water content was fixed during the NPT simulations. They therefore represent closed systems where water exchange with the surrounding environment does not occur. There is the potential for water to be expelled from the system during compression^37^. To explore this possibility, we designed a third system (Fig. 6D) where a slab region 10 Å thick was defined in the z direction that spans the entire simulation box in the *x* and *y* directions of the final configuration of the *‘2 W’* system. All water molecules that moved into this region during the simulation were extracted from the system by deletion. Note that water molecules can only move into this region if they can desorb from the nanoparticle surfaces and if there is pore connectivity. This extraction method was described in more detail in our previous study of methane release in kerogen nanopores^40^. This simulation was conducted for 89 ns, during which the water content was reduced from 37.2 wt% (i.e., that of *‘2 W’* system) to 6 wt%. The results obtained for this system are labeled as *‘2W_dewatering’*. Note that after 89 ns, water molecules were still able to move to the deletion region but at a very slow rate (i.e., for the last 1 ns, only 13 water molecules were deleted). Assuming that all water molecules would eventually be expelled from the system, we deleted the remaining water molecules and continued compressing the simulation box. The results reported for this system are labeled as *‘2W_dry’*.
